# Nurse’s experience working 12-hour shift in a tertiary level hospital in Qatar: a mixed method study

**DOI:** 10.1186/s12912-023-01371-0

**Published:** 2023-06-20

**Authors:** Bejoy Varghese, Chithra Maria Joseph, Adnan Anwar Ahmad Al- Akkam, Rida Moh’d Odeh A. M. AL-Balawi, Esmat Swallmeh, Kalpana Singh

**Affiliations:** 1 Neuroscience & Medical Department, In-Patient Services, Hamad General Hospital, Doha, Qatar; 2 Neuroscience Department, In-Patient Services, Hamad General Hospital, Doha, Qatar; 3 Neurosurgery, In-Patient Services, Hamad General Hospital, Doha, Qatar; 4 Neuroscience, Medical & Outpatient Department, Hamad General Hospital, Doha, Qatar; 5 Nursing Research, Hamad Medical Corporation, Doha, Qatar

**Keywords:** Nurses, Experience, 12-h shift work, Tertiary Care Center

## Abstract

**Background:**

The use of 12-h shifts for nursing staff has become common in many healthcare settings, including tertiary hospitals, due to its potential benefits such as reduced handover time and increased continuity of care. However, there is limited research on the experiences of nurses working 12-h shifts, particularly in the context of Qatar, where the healthcare system and nursing workforce may have unique characteristics and challenges. This study aimed to explore the experiences of nurses working 12-h shifts in a tertiary hospital in Qatar, including their perceptions of physical health, fatigue, stress, job satisfaction, service quality, and patient safety.

**Methods:**

A mixed method study design was applied consisting of a survey and semi-structured interviews. Data was collected from 350 nurses through an online survey and from 11 nurses through semi-structured interviews. Data was analyzed using Shapiro–Wilk test and the difference between demographic variables and scores were examined using Whitney U test and Kruskal- Wallis test. Thematic analysis was used for qualitative interviews.

**Results:**

The results from quantitative study revealed nurses perception in working 12-h shift has negative impact in their wellbeing, satisfaction as well as patient care outcomes. Thematic analysis revealed real stress and burnout and experienced an enormous amount of pressure going for work.

**Conclusions:**

Our study provides an understanding of the nurse’s experience working 12-h shift in a tertiary level hospital in Qatar. A mixed method approach informed us that, nurses are not satisfied with the 12-h shift and interviews revealed high level of stress and burnout among nurses resulting in job dissatisfaction and negative health concerns. Nurses also reported that it is challenging to stay productive and focused throughout their new shift pattern.

**Supplementary Information:**

The online version contains supplementary material available at 10.1186/s12912-023-01371-0.

## Background

In Qatar, the healthcare system is divided into three levels: primary, secondary, and tertiary. Tertiary level hospitals are the highest level of healthcare facilities in the country and provide highly specialized medical care, including complex surgeries and procedures, for patients with more severe or complicated health conditions.

These hospitals have advanced medical equipment and highly trained medical staff, including specialists in various medical fields, who can provide comprehensive and multidisciplinary care to patients. They also have advanced diagnostic capabilities, including laboratory and imaging services that allow for precise and accurate diagnosis of medical conditions.

In Qatar, Hamad Medical Corporation (HMC) is the main provider of tertiary level healthcare services, with six tertiary hospitals located throughout the country. Overall, tertiary level hospitals in Qatar play a critical role in providing advanced and specialized medical care to patients with complex health conditions, and they are essential components of the country's healthcare system.

Healthcare quality and patient safety can be affected by the environment of healthcare organizations. Longer working hours may be linked to work-related risks, nurses' health, and the caliber of their care [1]. The need for nurses to provide high-quality and optimum patient care has not changed despite the evolving healthcare systems [1, 2]. Numerous studies indicate that 12-h or longer shifts are becoming more typical for hospital nurses, but there is concern that these long shifts may negatively impact nurses' well-being, job satisfaction, and intention to leave their position. Long shift nurses are more likely to make mistakes that endanger patient safety and have unfavorable consequences [3]. Extended work hours can cause emotional weariness and burnout, both of which can impair the quality of nursing treatment [4]. Long work hours among nurses and other healthcare workers have been linked to burnout, lowered job satisfaction, and an increased chance of mistakes and unfavorable outcomes, according to studies [4].

Shift work and long hours are linked to higher prevalence of musculoskeletal illnesses, depression, anxiety, and cardiovascular disease in nurses [5]. Nursing staff have been shown to have poorer cognitive function and lower alertness after working consecutive long shifts without enough time for rest and recuperation [3]. These effects may jeopardize patient safety. Extended work hours and excessive workload are major contributors to nurse burnout, which can lead to negative patient and organizational outcomes [6]. Nurses working in Cambodian hospitals often exceed their mandated work hours, resulting in longer work weeks, increased susceptibility to burnout, and potential adverse effects on patient outcomes [7]. While working overtime is a common coping mechanism for nurse staffing shortages, it can also negatively affect the quality of patient care and the well-being of the nurses [4].

According to studies, fatigue brought on by insufficient sleep and lengthy workdays may be a factor in medical mistakes, workplace accidents, and car accidents [8]. Because it can affect patient safety, care quality, and nurse wellbeing, fatigue is a problem in nursing. Fatigued nurses are more likely to make medication mistakes, fail to recognize changes in a patient's condition, have low job satisfaction, and have high turnover rates. Poor attention, forgetfulness, poor decision-making, impatience, and poor contact with colleagues were among the most often mentioned consequences of exhaustion on nursing practice [3]. Fatigue was also seen to have detrimental consequences on nurses' wellbeing, resulting in stress, burnout, and low work satisfaction [4].

The levels of work satisfaction, burnout, and departure intention among nurses have been the subject of several research in the past [9]. Working hours remain a major factor that stands before all other factors because they determine not only an employee's physical well-being but also their family, social, and work-life balance [10]. Other factors include leadership style, adequate pay, pleasant working conditions, and good education and training facilities. Longer working hours will result in employees being less productive and unsatisfied with their jobs, which will contribute to job retention [11]. A study emphasized that long working hours can have adverse effects on safety and health, leading to increased risks, fatigue, and potential negative impacts on overall well-being [5]. A systematic review and meta-analysis, revealed a significant association between shift work and an elevated risk of vascular events, highlighting the importance of considering the impact of shift work on cardiovascular health. Nurses who work lengthy shifts or numerous consecutive shifts are more likely to get physically and mentally exhausted, which lowers job satisfaction and increases turnover rates [12].

Cutting the duration of work shifts and lengthening the period of time between shifts has been linked to decreased tiredness and increased job satisfaction among nurses [13]. Nurses who suffer negative workplace outcomes including burnout and job stress are more likely to experience poorer job satisfaction and higher turnover rates [14].

A cross-sectional survey [15] revealed that nurses in Hunan, China, experiencing job dissatisfaction and burnout, may face challenges in providing quality healthcare services, potentially impacting patient satisfaction and treatment outcomes.

Another study revealed that nurses working 12-h shifts were more likely to report lower job satisfaction, higher levels of burnout, and an increased intention to leave their current positions, highlighting the potential negative effects of longer shifts on nursing staff [16]. A study investigated the impact of verbal abuse on job stress among special unit nurses and general ward nurses in general hospitals, revealing that verbal abuse significantly contributed to increased job stress levels in both groups [17]. In an observational study, it was found that nurse staffing levels, work environments, and education significantly influenced patient mortality rates, highlighting the importance of these factors in healthcare outcomes [18].

In order to relocate workers away from the Hamad General Hospital (HGH), Doha (Qatar), for the newly established COVID facilities, a 12-h shift pattern was implemented beginning in April 2020. It has been noted that staff sick leave, overtime in leave absence, Hospital Acquired Pressure Injury, documentation deficits, job retention, and resignation gradually increase after the implementation of long working hours in HGH and its abrupt change from 40 to 60 h per week. Hence, despite rising interest in the study on nurses' experiences working 12-h shifts in HGH.

The purpose of this study is to assess nurses' perceptions of aspects such as exhaustion, stress, burnout, job satisfaction, physical health, documentation, and how long shifts affect the standard of nursing care.

## Methods

### Design

A mixed method cross-sectional study was adopted to explore inpatient nurses’ experiences in working 12-h shift that included a questionnaire to obtain quantitative data and semi structured interview to obtain qualitative data. This survey based descriptive research work have been used one of the Morgan’s practical strategies for combining research- preliminary quantitative and follow up qualitative research approach. The initial quantitative research guided us in purposive sampling and provided preliminary results that were then explored in greater depth through a complementary qualitative results. This mixed-methods approach is becoming more prevalent among survey researchers, as it allows for qualitative follow-up interviews to expand upon what was found from quantitative results. The use of multiple methods in a single research project may yield more comprehensive results than using each method independently [19–21].

### Setting and participants

The study was conducted in in-patient units of Hamad General Hospital, tertiary level hospital in Doha, state of Qatar. It involved 350 registered in-patient nurses delivering direct care to patients.

### Sampling method and recruitment

#### Quantitative study

**Fig. 1:**
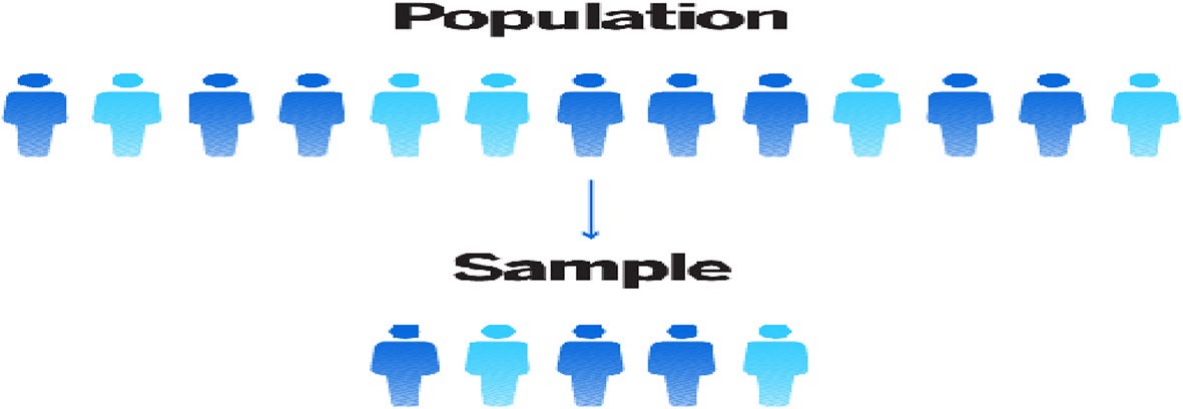
Simple random sampling

Simple random sampling was used, in every participant has a similar chance of being picked as the subject (Fig. 1). To analyze the perception of nurses regarding factors related to long working hours and the negative impacts of such long working hours, the mean and standard deviation were utilized. After obtaining approval from IRB of Hamad MRC, email was sent to nurse’s who fulfil the criteria for inclusion to participate in the research. The research permission form, MRC approval, research information sheet, and link to the online survey questionnaire were delivered to randomly selected participants via email along with a request for their interest in participating in an interview. Throughout the whole study, anonymity and confidentiality were ensured. Due to COVID-19 restrictions, face-to-face interviews was not successful for this study. MS Forms was used to design the survey questionnaire and gauge participant interest in participating in an interview. The survey was accessible for one month, and two email reminders were given. Within two weeks of the study's clearance in August 2021, 350 nurses responded.

#### Qualitative study

**Fig. 2:**
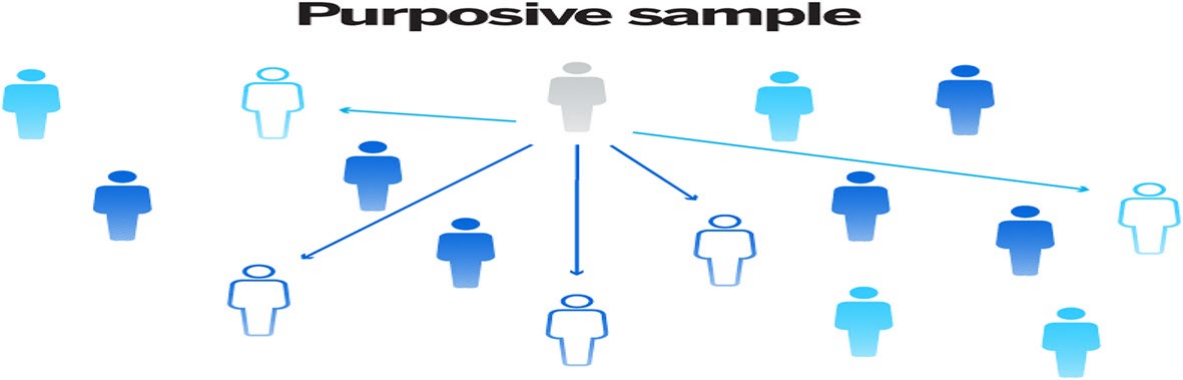
Purposive sampling

A purposive sampling method was used to select participants who had experienced working 12- hours. Purposive sampling involves selecting individuals who are likely to have valuable insights into the research topic, based on specific criteria (Fig. 2). In this study, the criteria for inclusion were based on nurses who had worked 12-h and who deliver direct patient care in Hamad General Hospital (HGH) as in-patients. The semi structured online interview sampled  20 inpatient nurse’s delivering direct patient care in Hamad General Hospital. According to Creswell, 1998, sample size guidelines suggested a range between 20 to 30 interviews to be adequate [22].

### Study tools

The study utilized a questionnaire based on Likert's five-point scale with options ranging from Strongly agree, Agree, Neutral, Disagree and Strongly disagree. The scale was normalized by assigning values of 2, 1, 0, -1, and -2 to the options, respectively. The questionnaire consisted of three sections.

Section 1 focused on gathering information about the profile of the respondents, Section 2 aimed to explore the perception of nurses towards long working hours, and Section 3 was designed to capture the experiences of nurses towards the impacts of long working hours on the quality of care.

To assess the validity and reliability of the questionnaire, a pilot study was conducted with 30 nurses. The internal consistency of the scale was measured using Cronbach’s alpha, with a reliability of 0.954. This indicates that the questionnaire is a reliable tool for measuring the perceptions and experiences of nurses towards long working hours and the impacts on the quality of care.

Qualitative interviews were done through MS teams. A semi structured one to one interview through MS Teams was conducted to collect the perception of nurses towards 12-h shift and the online one to one interview took place during nurses’ day off schedule to generate meaningful options, suggestions, and feedback. To ensure ethical standards, participants were provided with a research consent form that explained all aspects of the study, including their right to voluntary participation, withdrawal from the study at any time, and their right to privacy and confidentiality. Participants had the option to refuse involvement or omit answers from the questionnaire if they wished to do so. Interview was for 1 h and we encouraged participants not to mention their name. The whole audio was recorded through an option called “Start Recording” in MS Teams. 20 inpatient nurses were included in the online interview based on their voluntary interest. Experiences and perceptions are recorded and secured by principal investigator. The interview was stopped when we got data saturation in terms of answers. The 11 interviews were completed in the mid of September 2021.

Overall, the questionnaire and the qualitative interviews were designed and conducted to ensure the validity and reliability of the data collected and to maintain ethical standards throughout the study.

### Study size

The sample estimated 350 in-patient nurses delivering direct care to patients out of the total 3100 nurses in HGH based on 95% CI and 5% margin of error. The semi structured online interview sampled 20 inpatient nurse’s delivering direct patient care in Hamad General Hospital.

### Inclusion and exclusion criteria

The inclusion criteria for the study were all registered nurses who deliver direct patient care in Hamad General Hospital (HGH) as in-patients. This means that all RNs who work in HGH were responsible for providing direct care to patients during their hospitalization are eligible to participate in the study.

Exclusion criteria were nurses who have been orientees’ or preceptees for less than 3 months at HGH will not be included in the study. Additionally, patient care assistants (PCAs), nurses without a valid license, and nurse managers was also be excluded from the study.

### Data analysis

Descriptive statistics was used in this study to summarize data from the sample utilizing indexes, including means, SD, Median, IQR percentages, and frequencies. The normality assumptions of scores were assessed using the Shapiro–Wilk test. To examine the difference between demographic variables and scores, the Mann–Whitney U test and Kruskal–Wallis test were used.

STATA 17.0 software was used for all statistical analyses, and all tests were two-sided, with a *p*-value < 0.05 considered significant.

In this study, the thematic analysis framework described by Braun and Clarke [23] was used to guide the qualitative data analysis of in-depth interviews conducted with in-patient nurses. To ensure ethical standards, participants were provided with an informed consent form and their anonymity was maintained throughout the study. To confirm the trustworthiness of qualitative data, researchers often employ strategies such as member checking, researcher reflexivity, peer debriefing, and the use of verbatim quotes [24].

The verbatim was transcribed using the "Transcription" option in MS Teams and qualitative data was analyzed based on two categories: unintended consequences of adopting a 12-h shift pattern and factors influencing responses to a 12-h shift pattern. The researchers used a framework analysis approach and data coding was done using both deductive and inductive approaches. Two researchers (BV and CJ) independently reviewed and coded the transcripts and identified initial themes, with a third researcher (AA) helping to develop consensus around themes and addressing disagreements between the initial coders. After reconciliation, the final codebook was revised and clusters of linked codes were grouped into categories, emergent themes, and verbatim quotes. Atlas ti software version 8 was used for the data analysis.

### Data quality assurance

The credibility, dependability, transferability, and confirmability of the findings were enhanced through the application of four criteria. Credibility was achieved by engaging participants for an extended period and utilizing the researcher's professional experience. Furthermore, constant analysis of the qualitative data was conducted in conjunction with the review of the quantitative data to enhance credibility. Dependability was ensured by recording details of the research process, allowing for the replication of similar research. To ensure transferability and confirmability, detailed journal notes were kept, and an audit trail of all aspects of the research process was maintained.

### Trustworthiness

To enhance the validity and rigor of the qualitative data, several validation strategies were employed. These included sharing the findings with other researchers and participants to obtain feedback and validation (peer debriefing and member checking), ensuring transparency and reflexivity by addressing researchers' biases through constant self-reflection and recording in research memos, collecting data until saturation was reached to ensure comprehensive and representative findings, and providing detailed descriptions of the research context, methodology, and findings (thick description) to enhance the transferability of the findings to other settings or contexts. These strategies helped to ensure the credibility, dependability, transferability, and confirmability of the qualitative data, and ensured that the findings were robust and trustworthy.

To enhance the credibility of the quantitative data, 10% of the data was analyzed by two researchers. To enhance the credibility of the quantitative data, a process of inter-rater reliability was conducted. Specifically, 10% of the data was randomly selected and analyzed independently by two researchers to ensure consistency in coding and data interpretation. The inter-rater reliability score was calculated using the Cohen's kappa coefficient, which measures the agreement between the two raters beyond chance. A score of 0.8 or higher indicates excellent agreement, 0.6 to 0.8 indicates good agreement, and below 0.6 indicates poor agreement. In case of any disagreements, the researchers discussed and reached a consensus on the appropriate coding. The inter-rater reliability score achieved was 0.85, indicating excellent agreement. This approach increases the rigor and validity of the research findings, as it minimizes the risk of biases and errors in data interpretation.

## Results

The quantitative and qualitative findings were arranged according to the sections. In addition, the qualitative findings explored challenges faced during the 12-h work shifts.

### Participant characteristics

Out of the 350 nurses, the majority of the nurses responded from surgical specialty was 144(41%) followed by nurses from medical 123 (35%), ICU 38(10.9%), pediatrics 26(7.4%), emergency department 17(4.9), dialysis 1(0.3%) and operating theatre 1(0.3%).

The researchers collected demographic and work-related information from the participating nurses, and these details were presented in Table 1. The data revealed that the average age of the staff nurses was 35.7 ± 6.5 years, with the majority of them being female (84.3%). Regarding their educational qualifications, 83% of the nurses held a BSN, 14.5% had a GNM, and only 2.3% had a master's or doctorate degree. Most of the nurses were unmarried (81.7%). In terms of work experience, 49.4% of the nurses had 1 to 5 years of experience, 25.1% had 6 to 10 years of experience, 11.7% had 11 to 15 years of experience, 9.4% had 16 to 20 years of experience, and only 4.3% of the nurses had more than 20 years of experience.

**Table 1:** Participant characteristics

	Level	N (%)
N		350
Gender	Male	55 (15.7%)
	Female	295 (84.3%)
Age, mean (SD)		35.7 (6.5)
Marital status	Married	64 (18.3%)
	Single	286 (81.7%)
Educational qualification	BSN	291 (83.1%)
	GNM	51 (14.6%)
	MSN	7 (2.0%)
	PHD	1 (0.3%)
Years of work experience in HMC	1–5	173 (49.4%)
	11–15	41 (11.7%)
	16–20	33 (9.4%)
	6–10	88 (25.1%)
	> 20	15 (4.3%)
Fatigue, mean(SD)		2.4 (5.8)
Fatigue, Median (IQR)		3.0 (-3.0, 7.0)
Stress & burn out, mean (SD)		1.5 (5.1)
Stress & burn out, Median (IQR)		1.0 (-3.0, 5.0)
Job satisfaction, mean (SD)		1.4 (2.8)
Job satisfaction, Median (IQR)		1.0 (0.0, 3.0)
Physical health, mean (SD)		1.4 (4.9)
Physical health, Median (IQR)		2.0 (-2.0, 5.0)
Documentation, mean (SD)		3.4 (4.1)
Documentation, Median (IQR)		3.0 (0.0, 6.0)
Service quality, mean (SD)		0.1 (4.1)
Service quality, Median (IQR)		1.0 (-2.0, 3.0)
Patient safety, mean (SD)		1.8 (2.9)
Patient safety, Median (IQR)		1.0 (0.0, 4.0)
Adverse event, mean (SD)		2.6 (4.2)
Adverse event, Median (IQR)		2.0 (0.0, 5.0)
communication, mean (SD)		2.2 (2.5)
communication, Median (IQR)		2.0 (1.0, 3.0)

The study found that out of the 11 interviewees, 9 nurses found difficulty working the 12-h shift while 2 suggested continuing with the new shift pattern. The interview lasted for almost 1 h. The verbatim statements were recorded under the relevant themes. The participants included 2 male and 9 female RNs working in inpatient units of Hamad General Hospital. The RNs were named RN1 to RN11 to maintain anonymity and confidentiality. All says, they never compromised the patient safety for any reason, but the quality of care delivered is less in the 12-h shift time.

Seven main themes emerged from the data, namely (i) Health and work balance (ii) Family separation and stress (iii) Dissatisfaction with job (iv) Documentation burden and care (v) Productivity and quality care (vi) Patient safety priority (vii) Continuing education and feedback.

#### Health and work balance

The overall mean physical health score was 1.4 ± 4.9 and the mean of fatigue score was 2.4 ± 5.8 (Table 1). GNM nurses had a significantly higher fatigue score compared to BSN and MSN/PHD nurses, with median scores of 5.0 (1.0, 9.0), 3.0 (-3.0, 8.0), and 4.5 (-4.5, 6.5) respectively, with a *p*-value of 0.05 (Table 2).

**Table 2:** Association between demographic characteristics and fatigue, stress and burnout, job satisfaction, physical health and documentation scores

		Fatigue	Stress & Burn out	Job satisfaction	Physical health	Documentation	Service quality	Patient safety	Adverse Event	Communication
	N	Median (IQR)								
**Gender**										
Male	55	2.0 (-3.0, 8.0)	0.0 (-4.0, 4.0)	1.0 (0.0, 4.0)	0.0 (-4.0, 5.0)	4.0 (1.0, 7.0)	2.0 (-2.0, 5.0)	1.0 (-1.0, 4.0)	3.0 (-1.0, 5.0)	2.0 (1.0, 4.0)
Female	295	4.0 (-3.0, 8.0)	2.0 (-3.0, 6.0)	1.0 (0.0, 3.0)	2.0 (-2.0, 5.0)	4.0 (1.0, 7.0)	1.0 (-3.0, 3.0)	2.0 (0.0, 4.0)	3.0 (0.0, 6.0)	2.0 (1.0, 3.0)
*p*-value		0.4	0.15	0.84	0.2	0.8	0	0.42	0.76	0.4
**Marital Status**										
Married	64	2.0 (-3.5, 6.0)	2.0 (-2.5, 7.0)	1.0 (-1.0, 3.0)	1.5 (-3.5, 5.0)	4.5 (2.0, 7.0)	1.0 (-3.0, 2.0)	2.0 (0.0, 4.0)	4.0 (1.5, 6.0)	2.0 (1.0, 3.0)
Single	286	4.0 (-3.0, 8.0)	1.0 (-3.0, 5.0)	1.5 (0.0, 3.0)	2.0 (-2.0, 5.0)	4.0 (1.0, 6.0)	1.0 (-2.0, 3.0)	2.0 (0.0, 4.0)	2.0 (-1.0, 5.0)	2.0 (1.0, 4.0)
*p*-value		0.1	0.33	0.15	0.57	0.1	0.2	0.45	0.01	0.36
**Educational Experience**										
BSN	291	3.0 (-3.0, 8.0)	1.0 (-3.0, 6.0)	1.0 (0.0, 3.0)	2.0 (-3.0, 5.0)	4.0 (1.0, 6.0)	1.0 (-3.0, 3.0)	2.0 (0.0, 4.0)	3.0 (0.0, 6.0)	2.0 (1.0, 3.0)
GNM	51	5.0 (1.0, 9.0)	2.0 (-3.0, 6.0)	2.0 (0.0, 5.0)	2.0 (-1.0, 5.0)	5.0 (1.0, 7.0)	1.0 (-3.0, 3.0)	2.0 (0.0, 4.0)	2.0 (-1.0, 6.0)	2.0 (0.0, 4.0)
MSN/PHD	8	4.5 (-4.5, 6.5)	0.0 (-2.5, 4.0)	1.5 (-0.5, 3.0)	-0.5 (-5.0, 3.5)	2.5 (1.5, 5.5)	2.0 (0.0, 3.5)	1.0 (-1.0, 1.0)	1.0 (-0.5, 3.5)	3.0 (3.0, 4.0)
*p*-value		0.1	0.76	0.31	0.35	0.4	0.6	0.25	0.36	0.17
**Years of experience**										
1–5	173	3.0 (-3.0, 7.0)	1.0 (-3.0, 5.0)	1.0 (-1.0, 3.0)	2.0 (-3.0, 5.0)	3.0 (1.0, 6.0)	1.0 (-3.0, 3.0)	2.0 (0.0, 4.0)	3.0 (0.0, 6.0)	2.0 (1.0, 3.0)
11–15	41	2.0 (-5.0, 6.0)	-1.0 (-4.0, 6.0)	1.0 (0.0, 3.0)	1.0 (-3.0, 3.0)	4.0 (1.0, 7.0)	2.0 (-3.0, 3.0)	1.0 (-1.0, 3.0)	2.0 (-2.0, 5.0)	2.0 (1.0, 3.0)
16–20	33	5.0 (-2.0, 8.0)	3.0 (-2.0, 5.0)	2.0 (-1.0, 3.0)	1.0 (-2.0, 5.0)	3.0 (0.0, 7.0)	1.0 (0.0, 3.0)	3.0 (0.0, 5.0)	3.0 (0.0, 6.0)	3.0 (1.0, 5.0)
6–10	88	5.0 (0.0, 8.5)	2.0 (-2.0, 7.0)	1.0 (0.0, 3.0)	2.5 (0.0, 4.5)	5.0 (2.0, 7.0)	0.0 (-2.5, 2.0)	2.0 (0.5, 4.0)	3.0 (0.5, 5.0)	2.0 (1.0, 3.0)
> 20	15	3.0 (-5.0, 8.0)	3.0 (-5.0, 6.0)	2.0 (0.0, 4.0)	0.0 (-4.0, 5.0)	3.0 (-1.0, 9.0)	3.0 (-3.0, 5.0)	1.0 (-2.0, 5.0)	2.0 (-3.0, 6.0)	2.0 (1.0, 4.0)
*p*-value		0.1	0.47	0.79	0.52	0.2	0.3	0.56	0.5	0.61

The participants described various negative effects on their physical and mental health due to working 12-h shifts.

The verbatim from participants suggest that 12-h shifts can be physically and mentally exhausting, leading to skipped breaks, poor eating habits, and lack of sleep.

*RN3 *reported* a loss of weight due to irregular eating patterns, indicating the need for proper breaks and healthy eating habits.*

Nurses reported struggling to balance their work and personal lives, and some even experienced negative health outcomes as a result of the demands of 12-h shifts.

*“Sometimes I won't be taking two breaks. I won't be, I might be skipping one. I know it will affect my physical health.” (RN11)*

*“I don’t have enough sleep and healthy diet and my recent blood report shows there is some pre-diabetic stage.” (RN4)*

#### Family separation and stress

The overall mean of stress and burnout score was 1.5 ± 5.1. Stress and burnout score was not statistically associated with the demographic variables.

The 12-h shift has a significant impact on the stress and burnout of nurses. Participants expressed how the long working hours and staff shortage affected their mental health and family life. RN1 and RN4 shared their experience of working for long hours and not having enough time to spend with their family, causing them to feel stressed and frustrated. On the other hand, RN5 believes that teamwork can help manage stress and burnout, and thus prefers the 12-h shift.

*“I did not go for *vacation* to India, I couldn’t see one of my children for 2 years. So really is stressful for me and frustrated with this new shift pattern.” (RN4)*

#### Dissatisfaction with job

The overall mean score of job satisfaction was 1.4 ± 2.8 (Table1). Nine participants expressed their dissatisfaction with their jobs due to the 12-h shift, which they found to be exhausting and stressful, leading to a lack of job satisfaction and personal satisfaction. The long work hours, work overload, and lack of energy are leading to feelings of exhaustion, stress, and dissatisfaction with the job.

"There is a difference between job satisfaction and personal satisfaction. In 12-h shift I feel that I have not cared for the patient and as I am not energetic."(RN6)

"I want to quit the job because it is very hectic for me because I can’t concentrate on my family as well as I am not satisfied with the patient care with the present shift pattern."(RN8)

RN10 comments "I don’t feel fulfilled with my work anymore. It’s just work, and I don’t think I’m making a difference in the way I used to."

#### Documentation burden and care

The overall mean score of documentation was 3.4 ± 4.1. We didn’t find any statistical association between documentation challenges score and demographic variables.

The participant’s express frustration about the amount of documentation required during the shift, which they feel takes away from the time they could be spending on patient care. The technical and system errors also contribute to the delay in completing documentation. Additionally, the discrepancy between the required 8-h documentation and the 12-h shift adds to the burden of documentation.

RN4 says “Two times documentation during 12-h shift is time consuming and more focus is on documentation rather than delivering routine care.”

#### Productivity and quality care

In terms of service quality, the overall mean score was 0.1 ± 4.1. In terms of gender, male 2.0 (-2.0, 5.0) have higher quality services as compares to female 1.0 (-3.0, 3.0) which is statistically significant *p* = 0.029.

The verbatim from RN3, RN4, and RN7 highlight the impact of 12-h shifts on the quality of care delivered. RN3 suggests that care performance is highly effective during the initial couple of hours, but the quality of care may decrease as fatigue sets in. RN4 states that the ratio of patient care is more in 12-h shifts, leading to exhaustion while positioning patients, resulting in lower quality care. RN7 believes that good quality care can be delivered in 8-h shifts and that longer shifts make it difficult to attend educational sessions.

The above experiences suggest that the quality of care delivered in 12-h shifts may be affected due to fatigue, increased workload, and the inability to attend educational sessions. Therefore, it is important to find a balance between the need for longer shifts and the ability to provide quality care.

*"Care performance or the productivity of the care is highly effective because our mind is fresh and not tired during the initial couple of hours, but we try to deliver the high quality of care."(RN3)*

#### Patient safety priority

The overall mean of patient safety score was 1.8 ± 2.9 and the adverse event mean score was 2.6 ± 4.2.

The results showed that married nurses had a significantly higher median score of adverse events compared to single nurses, with a median score of 4.0 (1.5, 6.0) and 2.0 (0.0, 4.0) respectively, with a *p*-value of 0.009.

The perceptions of the participants highlight the issue of patient safety during the 12-h shift. RN2 acknowledges the possibility of errors, particularly medication errors, during the longer shift. However, RN2 also stresses that patient safety is never compromised. RN3 reports that no incidents such as pressure injuries, near misses or sentinel events were documented during the 12-h shift. This suggests that despite the challenges of the longer shift, patient safety was maintained.

*RN2 told “There is chance for many errors like medication errors during 12-h shift, but the patient safety is never compromised”*

### Continuing education and feedback

The overall mean score of communication was 2.2 ± 2.5. We didn’t find any statistical association between communication score and demographic variables.

The perceptions from RN3 and RN7 highlight the importance of communication and education in the workplace. RN3 observed that the 12-h shift pattern has resulted in diminished communication and personal interaction among staff, which can have negative impacts on the overall work environment. On the other hand, RN7 appreciated the frequent educational sessions and updates on errors and their action plan.

Effective communication is essential for creating a positive work environment and promoting patient safety. Regular educational sessions can keep the staff updated on best practices and help prevent errors. By prioritizing communication and education, health care organizations can improve the quality of care provided to patients.

*"I used to get very frequent classes and I am informed about the errors happened in the unit and its action plan." (RN7)*

## Discussion

The study aimed to explore the experiences of nurses working 12-h shifts in a tertiary hospital in Qatar, including their perceptions of physical health, fatigue, stress, job satisfaction, service quality, and patient safety. The findings of the study highlighted that nurse who worked 12-h shifts or longer, reported burnout, job dissatisfaction, and dissatisfaction with work schedule flexibility, and intention to leave their jobs. These findings are consistent with previous studies that have demonstrated the adverse effects of long working hours on nurses. The study also found an association between long working hours and adverse nurse outcomes. Working in a 12-h shift significantly affects the physical and psychological health and wellbeing of nurses, with adverse effects such as cognitive anxiety, fear, poor sleep quality, and musculoskeletal disorders being strongly associated with this shift system. Nurses reported insufficient time to eat and frequently skipped meals, which had a negative impact on their diet, nutrition, and health-related conditions.

Several studies have investigated the impact of shift patterns and work hours on nurse outcomes, including patient safety, job satisfaction, and burnout. For example, a study found that nurses working 12-h shifts had a higher risk of needle stick injuries compared to those working 8-h shifts [25]. Another study reported that nurses working 12-h shifts had a higher risk of musculoskeletal disorders compared to those working 8-h shifts [26]. A research conducted revealed that excessive overtime among nurses was associated with detrimental effects such as increased job dissatisfaction, higher burnout rates, and potentially compromised patient care quality [27]. A literature review conducted on hospital nurses' job satisfaction have revealed that working more than 40 h per week is associated with increased job dissatisfaction and potentially contributes to burnout among nurses [28].

Several studies have demonstrated that long work hours and overtime are consistently associated with shorter sleep duration and sleep disturbances [29]. A research indicated that nurses working extended shifts of 12 h or more experienced higher rates of medication errors and adverse patient events compared to those working shorter shifts [30]. Additionally, a study conducted found that prolonged work hours among nurses were associated with increased levels of emotional exhaustion and decreased job satisfaction, which can ultimately impact the quality of patient care provided [31].

Other studies have also investigated the impact of shift patterns on patient outcomes. For example, a study found that nurses working 12-h shifts were more likely to report medication errors compared to those working 8-h shifts [3]. Another study investigated the relationship between nurse staffing and patient falls on acute care hospital units, revealing that inadequate nurse staffing levels were associated with an increased risk of patient falls [32].

In addition to the studies mentioned above, several other studies have investigated the impact of shift patterns and work hours on nurse outcomes. For example, a study found that nurses working 12-h shifts had lower job satisfaction and higher burnout compared to those working 8-h shifts [33]. Another study reported that nurses working 12-h shifts had a higher risk of fatigue and decreased performance compared to those working 8-h shifts [34].

The scoping review exploring the association between nurse staffing levels and patient and nurses' outcomes in acute care hospitals across Japan revealed that inadequate nurse staffing levels were associated with negative patient and nurse outcomes, emphasizing the importance of appropriate staffing to ensure optimal care delivery [35]. Similarly, a study reported that nurses working 12-h shifts had lower scores for patient safety compared to those working 8-h shifts [36].

A study found a significant relationship between job satisfaction, professional identity, and the intention to leave the nursing profession among nurses in Turkey, suggesting that dissatisfaction and a weakened professional identity may contribute to the intention to leave the profession [37]. Furthermore, 12-h shifts may provide nurses with more free time and opportunities to fulfill family responsibilities, which can be a source of job satisfaction for those with families. According to a study, nurses working 12-h shifts reported experiencing difficulties in maintaining work-life balance, with limited time available for family responsibilities and reduced opportunities for meaningful engagement in family activities [38].

Another study investigated the impact of shift patterns on nurse outcomes in four European countries [39]. The study found that nurses working 12-h shifts had lower job satisfaction and higher burnout compared to those working 8-h shifts [39]. However, the study also reported that nurses working 12-h shifts had higher satisfaction with their work-life balance compared to those working 8-h shifts [39].

The findings of the study indicate that nurses working 12-h shifts experience adverse physical and psychological effects, which may lead to burnout, job dissatisfaction, and intention to leave the job. These findings are consistent with previous studies that have demonstrated the negative impact of long working hours on nurse outcomes. It is important to consider the potential advantages of 12-h shifts, such as providing nurses with more free time and opportunities to fulfill family responsibilities, but also to acknowledge their disadvantages, such as limited family time and reduced social activities. Therefore, further research is needed to examine the effects of 12-h shifts on nurses' job satisfaction and patient safety to provide evidence-based recommendations for shift systems in healthcare organizations. The findings of this study could be used by healthcare organizations to evaluate their current shift systems and consider alternative shift patterns to promote the health and wellbeing of nurses while ensuring patient safety.

In conclusion, the study highlights the importance of shift patterns in healthcare organizations and their impact on nurse outcomes. The findings suggest that 12-h shifts may not be suitable for promoting the health and wellbeing of nurses and may lead to adverse nurse outcomes. Healthcare organizations should consider alternative shift patterns to promote the health and wellbeing of nurses while ensuring patient safety. Further research is needed to examine the effects of shift patterns on nurse outcomes to provide evidence-based recommendations for shift systems in healthcare organizations.

The study conducted interviews with 11 nurses at Hamad General Hospital to understand their experiences while working 12-h shifts. The results of the study showed that while two nurses suggested continuing with the new shift pattern, the majority of the interviewees (9 nurses) found it difficult to work such long shifts. The data was analyzed thematically, and seven themes emerged from the interviews.

One of the critical themes that emerged from the data was related to the health and work balance of the nurses. The theme suggests that the nurses faced significant challenges related to their health and well-being while working 12-h shifts. This highlights the importance of considering the impact of long working hours on the well-being of healthcare professionals.

Another important theme that emerged was related to stress and burnout. The findings indicated that the nurses experienced high levels of stress and burnout while working the 12-h shift, which could potentially affect the quality of care provided to patients. This theme emphasizes the need to consider the impact of shift patterns on the job satisfaction and overall well-being of the healthcare professionals.

Furthermore, the theme of dissatisfaction with job was significant, as it indicates that the nurses had concerns about their job satisfaction and personal satisfaction while working 12-h shifts. This can affect the quality of care provided to patients and the overall well-being of the healthcare professionals.

The study also found that documentation burden and care were a significant concern for nurses working 12-h shifts. This emphasizes the importance of accurate documentation in ensuring patient safety and quality of care.

Additionally, the nurses perceived the quality of care provided to patients to be less during 12-h shifts, highlighting the need to consider the impact of shift patterns on the quality of care provided to patients. However, it is essential to note that the nurses did not compromise patient safety and it was their priority while working 12-h shifts, which is reassuring.

Finally, the theme of continuing education and feedback was also significant, as it indicates that the nurses faced challenges related to communication and education while working 12-h shifts. This theme highlights the need for effective communication and continuous education to ensure the delivery of high-quality patient care.

Overall, the study provides valuable insights into the challenges faced by nurses working 12-h shifts and highlights the importance of considering the impact of shift patterns on the well-being of healthcare professionals and the quality of care provided to patients. These findings can inform efforts to improve the work environment and patient care in healthcare settings.

### Strengths and limitations

This study was limited to only one facility of Hamad Medical Corporation. The measurement of patient outcomes through self-reporting by respondents may not necessarily provide a complete representation of adverse events. Future studies should aim to document the occurrence of adverse events and examine their potential correlation with working 12-h shifts.

Despite the limitations mentioned, this study provides valuable insights into the experiences of nurses working 12-h shifts in a hospital setting. The study's qualitative approach allowed for an in-depth exploration of the nurses' experiences and perspectives, providing rich data on the challenges they face. Additionally, the study's use of verbatim statements from the nurses themselves enhances the credibility of the findings and provides a nuanced understanding of the issues at hand. The emergence of seven distinct themes from the data further highlights the complexity of the issue and provides a comprehensive overview of the challenges faced by nurses working 12-h shifts. Overall, this study provides a valuable starting point for future research in this area and can inform efforts to improve the work environment and patient care in healthcare settings.

## Conclusion

This study highlights the challenges faced by nurses working 12-h shifts and emphasizes the need for healthcare organizations to prioritize the well-being of their staff. The findings suggest that these extended shifts can negatively impact nurses' physical and mental health, job satisfaction, and the quality of patient care. To address these issues, it is recommended that hospital managers consider shifting from 12-h shifts to 8-h shifts, recruit adequate nursing staff, and provide sufficient break times to mitigate the risks of fatigue and burnout.

These findings have implications for nursing education, emphasizing the importance of preparing future nurses to cope with the challenges of long working hours and make informed decisions about their preferred shift patterns. In clinical practice, hospitals and healthcare facilities should consider revising shift policies, prioritizing work-life balance and the well-being of staff, to enhance job satisfaction and promote better patient outcomes. In terms of research, this study adds to the body of literature on the impact of long working hours on healthcare professionals' well-being and patient care. Future research can build on these findings to explore the specific factors that contribute to the challenges faced by nurses working 12-h shifts and identify potential solutions to mitigate their negative impacts. Additionally, managers can implement policies that support staff well-being, such as flexible scheduling and opportunities for career growth.

Overall, this study contributes to the existing knowledge on the impact of extended shifts on healthcare professionals and patient care. By recognizing the challenges faced by nurses and taking proactive measures to improve their working conditions, healthcare organizations can create a supportive and sustainable environment that benefits both staff and patients.

## Supplementary Information

**Additional file 1: Supplementary file 1.** Interview guide.

## Data Availability

The availability of data and materials may be subject to certain access restrictions, such as ethical, legal, or commercial sensitivities. The Corresponding author Bejoy Varghese bejoy1987@gmail.com should be contacted if someone wants to request the data from this study.

## Abbreviations

COVID-19: Coronavirus disease 2019
HAPI: Hospital Acquired Pressure Injury
PS: Patient Safety; IRB: Institutional Review Board
MRC: Medical Research Center
HGH: Hamad General Hospital
