# Supplementary material for: Nurse’s experience working 12-hour shift in a tertiary level hospital in Qatar: a mixed method study
Source: BMC Nurs. 2023 Jun 20;22:213. doi: 10.1186/s12912-023-01371-0 (PMC10280874; doi:10.1186/s12912-023-01371-0)
Supplement: Supplementary file 1 — Additional file 1: Supplementary file 1. Interview guide. [file 12912_2023_1371_MOESM1_ESM.docx]

**Supplementary file 1: Title Interview Guide**

**A. Introductions**

Introduce moderator and co‐moderator and their role in this study research

**Moderator**: The aim of this study is to assess in-patient nurse’s experiences working 12-hour shift in Hamad General Hospital, Doha, and State of Qatar. The study will explore the nurse’s individual experiences and impact of long working hours on nurse’s health and quality of patient care. A mixed method study design is adopted, and the data will be collected from **350 nurses** through an online survey and a structured one to one interview will be conducted online through MS Teams among **20 nurses** working 12-hour shift in Hamad general hospital. Study evaluates the impacts of long working among nurses because long shift patterns is an important factor in determining well-being, satisfaction among nurses and patient care outcomes. Data will be synthesized in five categories such as nurse’s fatigue, stress and burnout, job satisfaction, physical heath, documentation, and quality of care.

**Moderator:** Thank you for agreeing to take part in this face-to-face interview. We the team appreciates your willingness to participate. The anticipated outcome of this study is to understand the challenges of staff nurses to deliver quality care in the workplace and deploy strategies and programs to promote wellness among nursing staff. In inpatient units of HGH, this approach will translate into improvements in workplace satisfaction and the quality of care provided by the nursing staff. Structured interviews are conducted with 20 nurses delivering direct care who had experience of working 12-hour shift in Hamad General Hospital. The nurses who wish to participate in interview were recruited through the link provided during the study.

**B. We have a few guidelines and rules to facilitate our interview:**

1. We want you to do explore your feelings on below questions. I may ask you to repeat if I haven’t heard from you in a while.

2. There are no right or wrong answers. Every person’s experience and opinion are important. We expect and want to hear a wide range of opinions and we do not anticipate consensus, just sharing.

3. We emphasize that what is said in this room should remain here. You should be comfortable to share anything if sensitive issues come up.

4. The interview will last for about one hour. Please silence your mobile phones.

We are only here to assist in this interview.

5. We will record this session as we want to capture everything you have to say. We don’t identify anyone by name in our findings. When you respond, be sure to not mention your name. You will remain anonymous. Your experiences and perception will be recorded and secured by the PI, Mr. Bejoy Varghese. Keep the video option on/off. We can provide summary details once the study is complete.

**C. Interview Questions**

1. What is your role as Health Care Worker in Hamad General Hospital?
2. What is your specialty of work?
3. How regularly you work 12-hour shift and for how long have you been working that

Pattern? What other shift patterns have you worked in the past?

1. What do you feel, considering fatigue when working 12-hour shift?
2. What is your perception on your stress and burnout when working in 12-hour shift?
3. What is the impact of working 12-hour shift on your physical health?
4. How do you consider the patient care documentation in working 12-hour shift?
5. What is your perception on impact of 12 hour shifts on job satisfaction?
6. What is the impact of working 12 hour shifts on your ability to deliver good quality care to your patients?
7. What is the likelihood to have adverse events when working in 12-hour shift? What is your experience on that?
